# Development of a structured interview for the modified version of the Beth Israel Hospital psychosomatic questionnaire for alexithymia

**DOI:** 10.3389/fpsyt.2024.1356643

**Published:** 2024-08-02

**Authors:** Gen Komaki, Takanobu Baba, Toshiyuki Yoshida, Tatsuyuki Arimura, Yoshiya Moriguchi, Motonari Maeda

**Affiliations:** ^1^ Faculty of Medical Science, Fukuoka International University of Health and Welfare, Fukuoka, Japan; ^2^ Faculty of Psychology, Otemon Gakuin University, Osaka, Japan; ^3^ Department of Social and Clinical Psychology, Hijiyama University, Hiroshima, Japan; ^4^ Faculty of Humanities, Kyushu Lutheran College, Kumamoto, Japan; ^5^ Department of Behavioral Medicine, National Institute of Mental Health, National Center of Neurology and Psychiatry, Tokyo, Japan; ^6^ College of Art and Design, Joshibi University of Art and Design, Sagamihara, Japan

**Keywords:** alexithymia (TAS-20), affect regulation, personality, openness to experience (O), neurosis and psychosomatic symptoms, affect awareness, attachment, stress coping

## Abstract

**Background:**

An observer-rated questionnaire for alexithymia based on the original 17-item Beth Israel Hospital Psychosomatic Questionnaire for Alexithymia (BIQ) was developed by Sifneos in 1973 and modified into a 12-item version of BIQ by Taylor et al. in 1997. However, it has rarely been used in a clinical or research context and studies have not given satisfactory inter-rater reliability for the 12-item version.

**Objective:**

To develop a structured interview in Japanese for the12-item modified version of BIQ (m-SIBIQ) to determine the reliability and validity of the m- scores and its factor structure model.

**Methods:**

Ninety-two Japanese young adults were interviewed. The inter-rater reliability of the m-SIBIQ was assessed by exploratory factor analysis. For the concurrent and convergent validities, correlation analysis was done between the scores of m-SIBIQ and the self-reported questionnaires: 20-Item Toronto Alexithymia Scale (TAS-20), NEO Five-Factor Inventory (NEO-FFI), Emotional Empathy Scale (EES), Interpersonal Reactivity Index (IRI), Beck Depression Inventory-II (BDI-II), and State-Trait Anxiety Inventory (STAI). Goodness of fit of the structure model of the m-SIBIQ was evaluated using confirmatory factor analysis, and the results were examined through stepwise multiple regression analysis.

**Results:**

Good reliability was obtained for the total score of m-SIBIQ: Cronbach’s α.950 (p<.001) and ICC.75(p<.05). The validity of the factor structure was obtained by confirmatory factor analysis using covariance. The model of the alexithymia constructs was configured by the operative thinking (la pensée opératoire) and affect awareness components. The stepwise multiple regression analysis extracted the total score of m-SIBIQ as significantly, negatively correlated with the Openness to experience score of NEO-FFI and significantly, positively correlated with the emotionally chilly score of EES and the score of difficulty describing feelings (DDF) of TAS-20. There were no correlations between the m-SIBIQ and BDI-II scores.

**Conclusion:**

For Japanese young adults, the m-SIBIQ is a reliable and valid instrument for overcoming weaknesses of the self-reported procedures by bringing to light the alexithymia construct and principal dimensions.

## Introduction

“A limited ability in understanding, processing, or describing one’s own feelings is generally referred to as “alexithymia,” and literally means “no words for feelings.” P.E. Sifneos (1) introduced this concept from his observations of psychosomatic patients who had “deficits in identifying, describing, and working with their own feelings as well as difficulty in distinguishing between their feelings and bodily sensations of emotional arousal.” Nemiah, Freyberger, and Sifneos later refined the alexithymia construct to include “a paucity of a fantasy and operative thinking as well as difficulty identifying and describing feelings” (2, 3). More recently, alexithymia has been used with broader populations with common medical and psychiatric disorders (4).

Several self-report measures of alexithymia have been developed, and the twenty-item Toronto Alexithymia Scale (TAS-20), one of the most widely used inventories (5, 6), has been translated into various languages, including Japanese (7–9). It has a three-factor structure: “Difficulty identifying feelings (DIF), difficulty describing feelings to others (DDF), and externally oriented thinking (EOT).” TAS-20 has provided a reliable, valid method for measuring alexithymia in both research and clinical practice. However, TAS-20 has been criticized for not including items of imaginal processes that directly assess the construct describing fantasies in the original TAS-26 (10–12). In addition, there are debates about whether or not a self-report scale like TAS-20 should be used to assess a construct that involves impairments in self-awareness (10–14). TAS-20 scoring may be influenced by negative affectivity (15–17).

When TAS-20 was administered concurrently with the NEO-Five Factor Inventory to a large number of nonclinical subjects (n=2,188), the results identified two subgroups with high TAS-20 scores (18): one with high scores for difficulty identifying feelings (DIF) and neuroticism on NEO-FFI and the other with high scores for externally oriented cognitive style and low openness to experience of NEO-FFI. These results suggest that the total score on the TAS-20 should not be considered a unidimensional measure of alexithymia.

Based on the above findings and others, the main problem with the alexithymia construct has always been its measurement. Because there are currently no uniform methods for evaluating alexithymia in a clinical setting, we translated the twelve-item modified version of the Beth Israel Hospital Psychosomatic Questionnaire (M-BIQ) (19, 20) into Japanese: the original BIQ is the first objective alexithymia scale that was developed by Sifneos (2). M-BIQ is an observer-rated measure with 12 items: nine items of the original BIQ were eliminated and four new items added. Six items assess the ability to identify and verbally communicate feelings (i.e., affect awareness) and six items assess imaginal activity and externally oriented thinking (i.e., operatory thinking, or pensée opératoire). Subsequently, we developed our Structured Interview (SIBIQ) to supplement TAS-20 for use with patients with psychosomatic symptoms (7). Factor analysis of the SIBIQ extracted alexithymia and fantasy ability as significant. We then did a similar investigation of healthy people with no or fewer symptoms, which was necessary to newly develop a modified structure interview format for the Japanese version of the M-BIQ (m-SIBIQ) for a broader population.

An observer-rated interview method, the Toronto Structured Interview for Alexithymia (TSIA), was reported in 2006 by Bagby et al. (21). The alexithymia construct of the TSIA is composed of the three facets of TAS-20 (DIF, DDF and OT) with an additional facet, fantasy and other imaginal processes (IMP). Although the construct validity has been reported to be excellent (22, 23), it needs special training for valid scoring of the 24 items and requires controlling for negative affectivity (23).

Herein, we examine and confirm the reliability and validity of our Japanese version of the m-SIBIQ.

## Materials and methods

### Participants, interviewers, and procedure

#### Eligibility

The study targeted university students aged between 19 and 25, all of whom passed the general academic ability test for university entrance. They were majoring in academic courses such as physical and occupational therapy, speech-language pathology, and psychology. None had failed, so academically they were no less linguistically competent than the average person. At the beginning of a lecture to 486 students, we distributed an A4 sheet of paper giving a brief summary of the research: a-cooperation request form containing detailed information about the nature of the government funded-scientific research program to investigate affectivity. After asking them if they would volunteer to take part in our research for a participation reward of 5,000 yen, 220 agreed to participate, with 93 of them taking the interview and completing the self-reported questionnaires at their homes, then returning them at the time of their first interview. They were not scored by the interviewers at that time. One person who did not fill out the BDI-II section of the self-reported questionnaires was excluded from the study, leaving the data of 92 students available for analysis (age range 19-25 years, mean age 20.7 ± 1.1 years: 29 men, mean age 20.6 ± .9 years and 63 women, mean age 20.7 ± 1.2 years). None of the subjects had a major medical, neurological, or psychiatric disorder, including schizophrenia, a depressive disorder, or an anxiety disorder, confirmed by the Mini-International Neuropsychiatric Interview (MINI) (24, 25).

We used three different interviewers/raters, one with a doctoral degree in medicine who specializes in psychosomatic medicine and psychiatry and two with doctoral degrees in psychology who specialize in clinical psychology. They also have master’s degrees in clinical/counseling and considerable training and experience in diagnostic interviewing.

### Questions used in the structured interviews

#### Development of the structured interview for the Japanese version of the modified Beth Israel questionnaire

The interview we developed for the Japanese version of m-SIBIQ is a 12-item structured interview based on the modified Beth Israel Hospital Psychosomatic Questionnaire (M-BIQ) (3, 19, 26) that asks patients with physical or psychiatric symptoms to describe how they perceive their own symptoms. “The 12-item questionnaire of the m-BIQ as originally reported is composed of two subscale scores: (a) *Affect Awareness* includes six items (items 2, 3, 5, 7, 9 and 12) that pertain to the ability to identify and verbally communicate feelings; and (b) *Operatory Thinking*, includes six items (items 1, 4, 6, 8, 10 and 11) that pertain to imaginal activity and externally oriented thinking” (3). Because the participants were non-patients with no symptoms, we modified the questionnaire protocol of the SIBIQ (19) by adding questions about feelings in response to negative life events they had experienced: bad, sad, difficult, stressful. If they replied that they had no such life events, we added “if” questions in which they were asked to imagine situations that would generally be expected to cause emotional responses (similar to the Alexithymia-provoked response questionnaire) (27) and asked them to answer in terms of their own emotions. The interviewers rated their answers on the same scale that is used for the m-SIBIQ, with the testers rating their answers according to our interview guidelines.

We request the interviewees to talk freely about their current complaints and symptoms. If they report past symptoms, we ask them to identify the life events that might be associated with their onset and/or exacerbation and ask how they feel about the events. The questions are specific, but if the interviewee does not understand a question, we rephrase it in such a way that they answer in their own words without being influenced by the interviewer; the interviewer adding concrete questions until the necessary information for rating is obtained. Interview guidelines and sample questions are available in the **Supplementary Material**.

#### Rating criteria

The explanation of an interviewee’s behavior should not be influenced by the interviewer’s own feeling or affect. Expressions that the interviewer considers to be unnatural: a policy, an official stance, and/or not the interviewee’s real feeling: are rated as inappropriate. The rating scale of the *m-SIBIQ* is a 7-point Likert type from 1 (*strongly disagree*) to 7 (*strongly agree*). Of the 12 items, 3, 7, 8, and 12 are negatively keyed such that scores on these items are appropriately converted before statistical analysis. The total score ranges between 12 and 84 points, with a higher score indicating a person is more alexithymic (2, 19).

Our structured interviews were always done by a pair of interviewers, both of whom have clinical experience with alexithymia. One was always G. K. Briefly, each subject was interviewed for approximately 20 ~ 30 min by one of the two interviewers, as randomly selected, but who in each interview asked the questions while the other watched in the interview room. After the interview, both scored the answers. When the score for a question was assessed differently, they decided on one or the other after discussing their reasons for giving the score.

#### The 20-item Toronto alexithymia scale

The Japanese translation of the 20-item Toronto Alexithymia scale (TAS-20) was done by Komaki, et al. (7) “TAS-20 is a self-reported questionnaire that consists of 20 items, with three subscales that measure the characteristics of alexithymia. Participants rate each question using a five-point Likert scale ranging from 1 (strongly disagree) to 5 (strongly agree). A higher score indicates that a person is more alexithymic. The TAS-20 includes three subscales: (a) difficulty in identifying feelings (DIF); (b) difficulty in describing feelings to others (DDF); and (c) externally-oriented thinking (EOT).” The Japanese version of the TAS-20 showed good reliability and validity in a large population (8).

#### NEO-FFI

“A five factor model of personality traits has been found to account for a large amount of the variance in the data from studies of personality” (28). “The NEO-FFI (NEO-Five Factor Inventory) consists of 60 items and is an abridged version of the NEO-PI-R (the Revised NEO Personality Inventory). It is a widely used self-report instrument designed to provide a general description of normal personality. The NEO-FFI uses a 5-point Likert scale, ranging from 0 (Strongly disagree) to 4 (Strongly agree). The five major domains (factors) of personality are as follows: Neuroticism (N), Extraversion (E), Openness to Experience (O), Agreeableness (A), and Conscientiousness (C). Scores are summed totals and have a range of 0–48 for each of the five personality domains.” The Japanese version of NEO-FFI has been well cross-validated and its reliability has been confirmed (29). High correlations (r = 0.82–0.92) between respective domains of the Japanese version of the NEO-PI-R and the NEO-FFI confirm that the two questionnaires have the same factorial structure.

#### Emotional empathy scale

The emotional empathy scale (EES) was developed by Mehrabian and Epstein (30) and the Japanese version was done by Kato and Takagi (31). It is a self-administered questionnaire that measures “emotional empathy,” defined as an “affective response to someone else’s emotional experience.” Mehrabian and Epstein (30) created the items of EES with the expectation of multiple subscales, but none were extracted. The Japanese version is subdivided into three components (31) for use with Japanese samples as follows: I) Emotional warmth; a tender and compassionate attitude toward other’s feelings. People with emotional warmth are impressionable in response to art, literature, and movies as well as with other’s sorrow and distress and sometimes participate in voluntary activities. 2) Emotionally chilly: an apathetic and sometimes disfavoring attitude toward other’s feelings like sorrow, distress, and joy. Such people always keep others at a distance. 3) Emotional affectedness; a tendency to be easily influenced by other’s feelings. It is almost the same as “emotional contagion.”

#### Interpersonal reactivity index

The interpersonal reactivity index (IRI) (32) is “a self-administered questionnaire that measures the empathetic ability of the participant. The Japanese version was developed by Aketa (33). The IRI consists of four scales, each measuring a distinct component of empathy: 1) empathic concern, feeling emotional concern for others: 2) perspective taking, cognitively taking the perspective of another; related to social competence. Factors (I) and (2) are characterized as desirable interpersonal styles: 3) fantasy, emotional identification with characters in books, films, etc.: and 4) personal distress, negative feelings in response to the distress of others.”

#### The Beck depression inventory-II

“The BDI-II is a 21-item self-report questionnaire used to assess the severity of depression symptoms that is based on the diagnostic criteria for depressive disorders in DSM-IV. Each item is scored from 0 to 3, with a higher score indicating greater intensity of the symptom. The total score is the sum of the items and rages from 0 to 63; a higher score indicates greater depression.” The reliability and validity of the Japanese version of the BDI-II are well documented (34).

#### State/trait anxiety scales

The Japanese version of the State-Trait Anxiety Inventory (STAI) was used to assess anxiety (35). STAI is a self-reported questionnaire consisting of two scales, with STAI-1 assessing state anxiety and STAI-2 assessing trait anxiety. Each scale consists of 20 items indicating the presence or absence of anxiety symptoms (36). The score of each scale ranges from 20 to 80.

### Statistical analysis

Data analyses were performed using IBM SPSS Statistics Ver. 27.0. and Amos Ver. 27.0 The level for **s**tatistical significance was set at p < 0.05.

## Results

### Reliability of the m-SIBIQ

For the sample data sets (n=92), the reliability of the m-SIBIQ was assessed from two perspectives: internal consistency by calculating Cronbach’s α and interrater reliability by calculating the intraclass correlation coefficient (ICC), which is used when there are more than two raters. Cronbach’s α was 0.950 (p<.001), indicating excellent internal consistency and the Intraclass Correlation Coefficient ICC (2,1) was 0.75 (p <.05), indicating good interrater reliability (37).

### Exploratory Factor Analysis of the m-SIBIQ

Exploratory factor analysis using the principal factor method with varimax rotation of the *samples* (n=92) was done to explore the factor structure of the *m-SIBIQ*. The sample performed adequately on the Kaiser-Meyer-Olkin measure (= 0.91 > minimum acceptable level = 0.50) and on the Bartlett’s test of sphericity (χ2 = 727.9, df = 66, P < 0.0001). Eigenvalues of 1, 2, and 3 number factors to be extracted were as follows: 9.15, 1.34, and 0.58, respectively. When we chose ‘eigenvalue >1’ criteria, the 2-factor solution was optimal with the theoretical constructability, and these two components accounted for 87.5% of the total variance. The two factors extracted, *Alexithymia* and *Fantasy Ability*, are different from the factors extracted from *the m-BIQ* (3). Items 1, 4, and 6, which were originally included in *Operatory Thinking*, are relegated to *Alexithymia* and items 8 and 10 are relegated to *Fantasy Ability*.

**Table 1A** shows the Pearson correlations of the total m-SIBIQ score for the self-reported questionnaires: TAS-20 Total and three Factors (DIF, DDF, EOT); NEO-FFI five factors; Emotional Empathy Scale (EES); Interpersonal Reactivity Index (IRI); State-Trait Anxiety Inventory (STAI); and BDI-II. Significant, positive correlations were obtained for TAS-20 Total and Factor 3 (EOT), Emotionally Chilly of EES, and State Anxiety, whereas significant, negative correlations were obtained for Extraversion (E), Openness to Experience (O), Agreeableness (A) of NEO-FFI, Fantasy of IRI, and Warmness of EES.

**Table 1A:** Pearson correlations of the m-SIBIQ total score with the self-reported questionnaires.

Self-reported Questionnaire		r	p	Self-reported Questionnaire		r	p
**TAS-20**	Total	233*	.025	**IRI**	Empathetic Concern	-.194	.064
	DIF	.179	.088		Fantasy	-.284**	.006
	DDF	.181	.084		Personal Distress	.050	.634
	EOT	.230*	.028		Perspective Taking	-.118	.264
**Neo-FFI**	Neuroticism (N)	.122	.245	**EES**	Emotional warmth	-.213*	.042
	Extraversion (E)	-.305**	.003		Emotionally chilly	.284**	.006
	Openness to Experience (O)	-.319**	.002		Emotional affectedness	-.034	.745
	Agreeableness (A)	-.218*	.037				
	Conscientiousness (C)	-.134	.204	**STAI**	State anxiety	.246*	.018
**Depression**	***Beck Depression Inventory-II (BDI-II)***	.072	.496		Trait anxiety	.158	.110

*p< 0.05, **p< 0.01.

**Table 1B** shows the Pearson correlations for the TAS-20 total score and its three Factors (DIF, DDF, EOT). All correlations are statistically significant (P < 0.01).

**Table 1B:** *Pearson correlations* for the Total score of TAS-20, DIF, DDF, and EOT score of the TAS-20.

	DIF	DDF	EOT
**Total TAS-20**	.915**	.857**	.633**
**DIF**		.718**	.371**
**DDF**			.347**
**EOT**			

**p< 0.01.

### Validity of the m-SIBIQ

#### Confirmatory factor analysis

**Figure 1:**
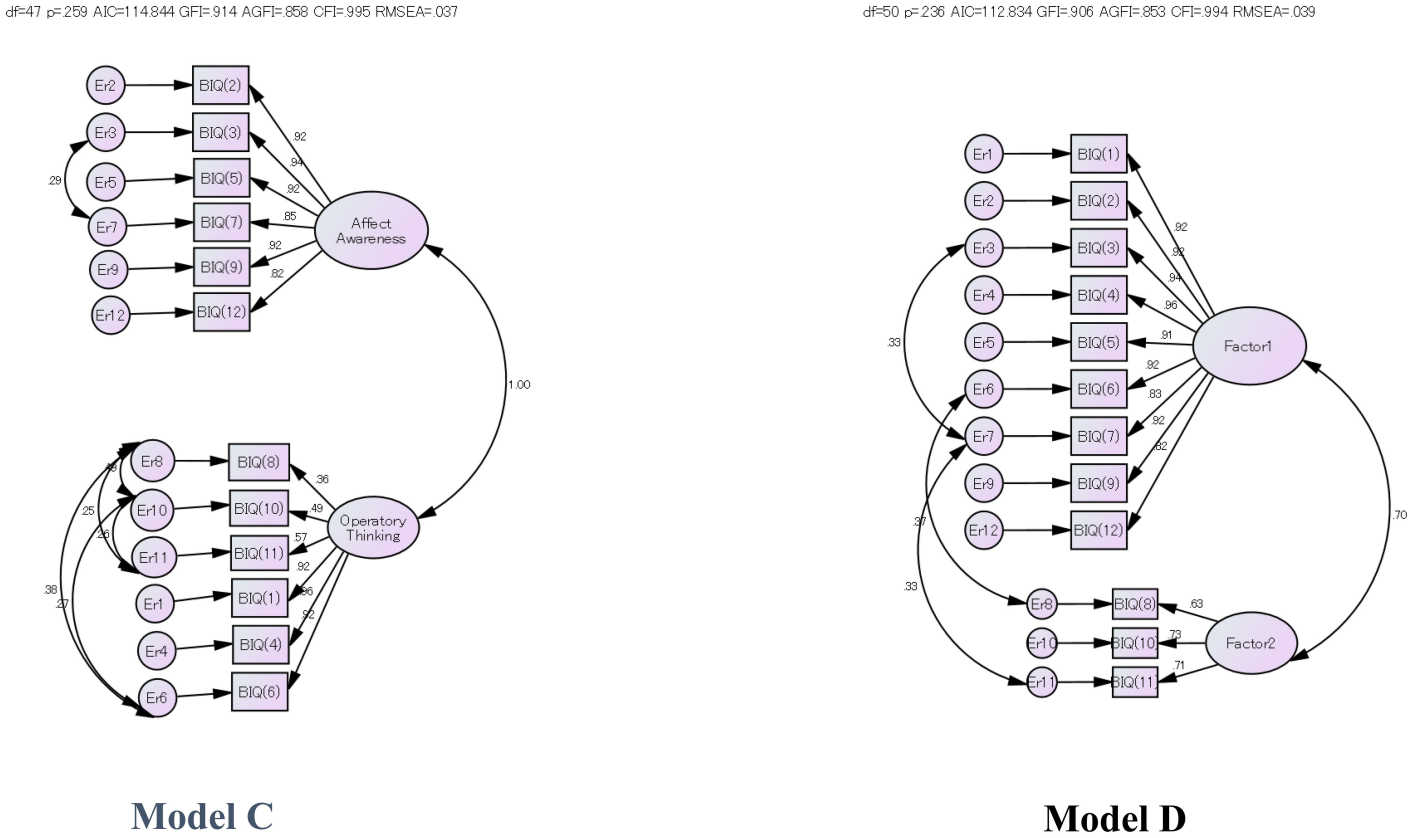
Various factor structure models for m-SIBIQ.

The two best candidate models are shown in **Figure 1**. We tested the goodness-of-fit results (N=92) for the candidates of the m-SIBIQ structure models. Although the sample number is too small to confirm the model structure, we further calculated the covariance as an error based on the exploratory factor analysis as well as the two-factor model of the original version of Sifneos, which allowed us to examine the goodness of fit of several hypothesized models. From among them, the typically good models of the candidates were examined, as described below:

Goodness-of-fit was evaluated by the following criteria as recommended by Cole and Marsh et al. (38, 39); goodness-of-fit (GFI) > 0.85, adjusted goodness-of-fit (AGFI) > 0.80, and root-mean-square residual (RMSR) <0.10. However, the GFI, AGFI, and RMSR are all dependent on sample size and tend to indicate a good fit in a large sample; thus, a good fit might have been obtained as an artifact of sample size, regardless of the real fit, in the present study. We also calculated the comparative fit index (CFI), root mean square error of approximation (RMSEA), and upper and lower end of the 90% confidence interval for the RMSEA to see if the interval includes the area of “close fit” at 0.05. The global fit indices are also supported by a RMSEA > 0.08 (preferably close model fit of < 0.06) and CFI > 0.90 (40).

**Model A**: Two factor model: the original two factor model by P.E. Sifneos composed of Affective Awareness (BIQ items 2, 3, 5, 7, 9 and 12) and Operatory Thinking (BIQ items 1, 4, 6, 8, 10 and 11). The fit of this model is not sufficiently high, assuming no covariances.

**Model B**: Two factor model: based on the results of the exploratory factor analysis; Alexithymia (BIQ items 1, 2, 3, 4, 5, 6, 7, 9 and 12) and Fantasy and Dream (BIQ items 8, 10 and 11).

**Model C**: Two factor confirmatory analysis model (Model A-1); Affect Awareness (BIQ items 2, 3, 5, 7, 9 and 12) and Operatory thinking (BIQ items 1, 4, 6, 8,10 and 11), assuming six covariances.

**Model D**: Two factor confirmatory analysis model (Model A-2); Alexithymia (BIQ items 1, 2, 3, 4, 5, 6, 7, 9 and 12) and Fantasy/Dream (BIQ items 8, 10 and 11), assuming three covariances (**Table 2**).

**Table 2:** C.F.A goodness of Fit: Various factor structure models for m-SIBIQ.

Model	CFA goodness of various factor structure models for m-SIBIQ.						
	^χ2 (d.f.)^	X^2^	AIC	GFI	AGFI	CFI	RMSEA
**A**	104.91(53)	0	154.91	0.843	0.768	0.958	0.104
**B**	82.92(53)	0.005	132.92	0.867	0.804	0.976	0.079
**C**	52.844(47)	0.259	114.844	0.914	0.858	0.995	0.037
**D**	56.834(50)	0.236	112.834	0.906	0.853	0.994	0.039

AIC, Akaike’s Information Criterion. GFI, goodness of fit. AGFI, adjusted goodness of fit. CFI, comparative fit index. RMSEA, root mean square error of approximation.

**Model A**: Original two factor model by P.E. Sifneos. Affect Awareness (BIQ items 2, 3, 5, 7, 9 and 12) and Operatory Thinking (BIQ items 1, 4, 6, 8, 10 and 11) assuming no covariances.

**Model B**: Two factor model: based on Principal component analysis (PCA), Alexithymia (BIQ items 1, 2, 3, 4, 5, 6, 7, 9 and 12) and Fantasy and Dream (BIQ items 8, 10 and 11).

**Model C**: Two factor confirmatory analysis model assuming six covariances in Affect Awareness (BIQ items 2, 3, 5, 7, 9 and 12) and Operatory Thinking (BIQ items 1, 4, 6, 8, 10 and 11).

**Model D**: Two factor confirmatory analysis model assuming three covariances in Alexithymia (BIQ items 1, 2, 3, 4, 5, 6, 7, 9 and 12) and Fantasy and Dream (BIQ items 8, 10 and 11).

### Stepwise multiple regression analysis

Multiple regression analysis was conducted to confirm the extent to which the characteristics of the self-reported questionnaire scales could explain the validity of the structure in each model (**Table 3**). The dependent variables were the total and two specific sub-class *m-SIBIQ* scores that were obtained by the Confirmatory Factor Analysis Models, and the independent variables were the scores of the self-reported questionnaires: three factors of TAS-20 (DIF, DDF, and EOT), five major domains of Neo-FFI (Neuroticism, Extraversion, Openness to experience, Agreeableness, and Conscientiousness), Emotional Empathy Scale (EES; Emotional warmth, Emotionally chilly, and Emotional affectedness), Interpersonal Reactivity Index (IRI; Empathic concern, Perspective taking, Fantasy, and Personal distress), BDI-II, and State/Trait Anxiety Scales. For the calculation formula, the TAS-20 total score was excluded beforehand because of its significantly high correlations obtained with the DIF, DDF, and EOT (**Table 1B**). Finally, the eighteen independent items above were used.

**Table 3:** Stepwise Multiple Regression Analysis of the dependent total and specific *m-SIBIQ* scores that were obtained by the Confirmatory Factor Analysis Models and the significantly related independent scores of the self-reported questionnaires.

Dependent variables, Factors		Independent variables								
Model		Item		Standardized β	t-score	*p*		adjusted R^2^	F value	*p*
**m-SIBIQ Total**		1	Openness (NEO-FFI)	- 0.359	- 3.785	0.001	**	0.092		
		2	Emotionally Chilly (EES)	0.270	2.855	0.005	**	0.172		
		3	DDF (TAS-20)	0.195	2.041	0.044	*	0.2	8.596	**
**C**	**Affective Awareness**	1	Extroversion (NEO-FFI)	- 0.321	- 3.351	0.001	**	0.094		
		2	EOT (TAS-20)	0.281	2.292	0.004	**	0.165	9.966	**
	**Operatory Thinking**	1	Openness (NEO-FFI)	- 0.287	- 2.901	0.005	**	0.114		
		2	Emotionally Chilly (EES)	0.226	2.399	0.019	**	0.171		
		3	Fantasy (IRI)	- 0.229	- 2.305	0.024	*	0.209	9.098	**
**D**	**Factor1 (alexithymia)**	1	Extroversion (NEO-FFI)	- 0.333	- 3.482	0.001	**	0.102		**
		2	EOT (TAS-20)	0.268	2.796	0.006	**	0.166	10.04	
	**Factor2 (fantasy/dream)**	1	Fantasy (IRI)	- 0.334	- 3.394	0.001	**	0.162		
		2	Openness (NEO-FFI)	- 0.247	- 2.505	0.014	*	0.209	12.99	**

*p <.05 **p <.01.

The results showed that the total m-SIBIQ model extracted one negative domain: Openness to Experience in NEO-FFI. No other personality domains were extracted. For other independent self-reported questionnaire items, Emotionally Chilly (EES) and DDF (TAS-20) were positive.

In **Model C**, the construct component Affective Awareness was negative for Extraversion (NEO-FFI) and positive for EOT (TAS-20), and the other construct component Operatory Thinking was negative for Openness to experience (NEO-FFI), positive for Emotionally Chilly (EES), and negative for Fantasy (IRI).

In **Model D**, the Factor 1 component “Alexithymia” was negative for Extraversion (NEO-FFI) and positive for EOT (TAS-20). For “Fantasy and Dreams” of Factor 2, both Fantasy (IRI) and Openness to experience (NEO-FFI) were negative.

## Discussion

The purposes of the present study were to develop a structured interview for alexithymia and to assess its reliability and validity for use with *m-SIBIQ.* The reliability, construct, and concurrent and discriminant validity of this *m-SIBIQ* were evaluated, and it was found to be reliable and valid for the assessment of alexithymia in Japan.

The total m-SIBIQ score model extracted one personality domain, a low score for Openness to Experience (NEO-FFI). Among the other independent items, high Emotionally Chilly (EES) and DDF (TAS-20) scores were extracted. Low Openness to experience and high Emotional Chill represent generally recognized characteristics of alexithymia (3, 41, 42). It is interesting that DDF rather than DIF was extracted as a typical TAS-20 subscale. It is said that the total TAS-scale is generally reliable, yet, the subscale scores can have an added value beyond the total scale score (42). The implication of this is that findings related to TAS DDF make a positive contribution to the m-SIBIQ total score, differently from DIF. TAS DIF was originally defined theoretically to distinguish between feelings and bodily sensations of emotional arousal (1), whereas DDF is related to social context like difficulties in social communication. This finding is consistent with Nemiah’s findings (43) that the alexithymia characteristics difficulties in ‘verbalizing’ and ‘analyzing’ represented by DDF and EOT scores have a significant negative relation with Openness to experience. In addition, Emotionally Chilly (EES) well reflects another characteristic of alexithymia that is related to uncooperative and critical interpersonal behaviors (44).

For construct validity, two best models, **C and D,** were obtained: **Model C** is composed of the facets Affective Awareness (AA) and Operative Thinking (OT); **Model D** is composed of Alexithymia (Factor 1) and Fantasy/dreams (Factor 2).

The facets of **Model C** are consistent with the construct characteristics of BIQ (42, 45) and confirmed the generally accepted structure model of alexithymia as described by Nemiah and Sifneos (2, 3). This construct model is composed of “Affective Awareness” and “Operative Thinking (la pensée opératoire)” components. For the lower facet of Affective Awareness, it extracts the personality trait Extraversion (NEO-FFI) as negative and the EOT factor of TAS-20 as positive. For the lower facet of the Operative Thinking style (la pensée opératoire), it extracts less Openness to Experience trait (NEO-FFI), a tendency toward Emotionally Chilly (EES), and less Fantasy (IRI) life. These findings are consistent with personality findings observed among people with alexithymic characteristics (42, 45).

In contrast, **model D** is distinguished by two facets of the alexithymia construct: *“Fantasy ability”* appears to be another independent factor related to “Alexithymia.” This model reconfirms lack of imagination as a concept of the alexithymia model (2), which was supported and measured in the original TAS but was later eliminated from TAS-20 because the validation of the ‘fantasy’ factor was not successfully established as an independent factor (20). Model D was observed previously in a patient group studied for the development of the SIBIQ (19). The facets fantasy and imaginal processes were poorly mapped on the alexithymia construct (45). The negative relation of the Extraversion personality trait to the alexithymia score is not surprising: an extrovert would have the ability to put feelings into words in the interviews, as when participating in a normal social context.

To summarize the findings above, a paucity of openness to experience as a personality trait significantly affects the formation of the total alexithymia characteristics for m-SIBIQ and strongly affects operatory thinking style and fantasy/dreams, which are characterized in the Alexithymia construct (46). The dimensions of personality traits in reports of recent studies are somewhat different from the present m-SIBIQ findings (12, 42, 45, 46). We completely agree with Rosenberg et. al’s concern that the differences are a limitation of almost all recent studies that examine the relation between alexithymia and the personality traits using self-reports like TAS-20 (47).

For discriminative validity, the depression scores of BDI-II were not correlated with *m-SIBIQ* in the present study. Many studies have shown that when using a self-reported alexithymia questionnaire like TAS-20, alexithymia appears to be associated with depression and anxiety (15–17, 20, 48). Particularly, the facet “difficulty identifying feelings” is related to negative affectivity, but alexithymia differs from depression. People who score high on the subscales of the above facets may be viewed by others as emotionally aware or they may use emotional language in relatively complex ways. Clearly, people with high negative affect differ substantially from those with alexithymia, who are much less interested in their own psychological and emotional lives. Moreover, this type of structured interview has less of an impact than self-evaluation, probably because when people are depressed they may talk less to others. Thus, one of the important findings of this study is that m-SIBIQ is not influenced by negative affectivity, such as depression.

Some reviews across numerous studies on alexithymia have shown a significant correlation with the neuroticism domain. The findings were from both TAS-20 and TAS-26 (10, 12, 46) which are self-reported questionnaires, thus the correlation of alexithymia with neuroticism may represent a method-specific effect (47). The present study of m-SIBIQ, however, supports evidence that, as Sifneos emphasized at every turn, “what is really needed is for physicians to learn to recognize the difference between neurotic and alexithymic patients” (1).

The present findings show a multi-dimensional view of the personality traits and personality type of persons with alexithymia (12). One reason for the absence of alexithymia in DSM-5 may be because of the failure of taxometric studies to identify alexithymia. As empirical evidence has suggested, alexithymia is related inversely to the psychological mindedness and emotional intelligence traits. Also, alexithymia is better thought of as a coping style that is used to defend against emotional distress associated with specific situations, such as trauma or chronic medical illness (49).

Attachment studies have reported an association between alexithymia and an insecure attachment life style in early childhood (50). Openness to experience may be necessary for the regulation of distressing emotions and doing something to change for the better the problem causing the distress (51). It should be emphasized that being open to one’s own feelings is essential. The relation of alexithymia to health and disease will be better clarified by development of the SIBIQ, and it will help with the investigation of various psychosomatic diseases and reduce the risk of their development.

Some limitations of the present study should be noted. First, our sample consisted of only young adults and there was a statistically small number of subjects. Second, all were well educated students, so more heterogeneous samples are necessary. Future research should include a patient group with high alexithymia scores and analysis of test-retest reliability to confirm these results.

The findings of the present study indicate that m-SBIQ is consistent with the most widely used assessments of the alexithymia construct and that it has great potential as a treatment and research tool for not only psychosomatic patients but also those who are difficult to treat in clinical settings. The characteristics identified represent the personality traits openness to experience, difficulty in describing feelings in self-reported expression, and less fantasy life.

## Data availability statement

The raw data supporting the conclusions of this article will be made available by the authors, without undue reservation.

## Ethics statement

The studies involving humans were approved by the ethics committee of International university of health and welfare. The studies were conducted in accordance with the local legislation and institutional requirements. The participants provided their written informed consent to participate in this study.

## Author contributions

GK: Writing – original draft, Writing – review & editing, Conceptualization. TB: Formal Analysis, Investigation, Methodology, Writing – review & editing. TY: Formal Analysis, Methodology, Writing – review & editing. TA: Conceptualization, Writing – review & editing. YM: Conceptualization, Writing – review & editing. MM: Conceptualization, Supervision, Writing – review & editing.

## References

[1] Sifneos PE. The prevalence of A’lexithymic’ characteristics in psychosomatic patients. Psychother Psychosom. (1973) 22:255–62. doi: 10.1159/000286529 4770536

[2] Nemiah JC Sifneos PD. Affect and fantsy in patients with psychosomatic disorders. In: Hill OW, editor. Modern trends in psychosomatic medicine, vol. 2. Butterworths, London (1970). p. 26–34.

[3] Nemiah JC Freyberger H Sifneos PE. Alexityymia: A view of the psychosomatic process. In: Hill OW, editor. Modern trends in psychosomatic medicine, vol. 3. Butterworths, London (1976). p. 430–39.

[4] Taylor GJ Bagby RM. New trends in alexithymia research. Psychother Psychosom. (2004) 73:68–77. doi: 10.1159/000075537 14767148

[5] Bagby RM Parker JD Taylor GJ. The twenty-item Toronto Alexithymia Scale–I. Item selection and cross-validation of the factor structure. J Psychosom Res. (1994) 38:23–32. doi: 10.1016/0022-3999(94)90005-1 8126686

[6] Bagby RM Taylor GJ Parker JD. The Twenty-item Toronto Alexithymia Scale-II. Convergent, discriminant, and concurrent validity. J Psychosom Res. (1994) 38:33–40. doi: 10.1016/0022-3999(94)90006-X 8126688

[7] Komaki G Maeda M Arimura T Nakata A Shinoda H Ogata I. The reliability and factorial validity of the Japanese version of the 20-Item Toronto Alexithymia Scale (TAS-20). Japanese J Psychosomatic Med. (2003) 43:839–46. doi: 10.5926/jjep1953.56.3_403

[8] Moriguchi Y Maeda M Igarashi T Ishikawa T Shoji M Kubo C. Age and gender effect on alexithymia in large, Japanese community and clinical samples: a cross-validation study of the Toronto Alexithymia Scale (TAS-20). Biopsychosoc Med. (2007) 1:7. doi: 10.1186/1751-0759-1-7 17371586 PMC1838425

[9] Nishimura H Komaki G Igarashi T Moriguchi Y Kajiwara S Akasaka T. Validity issues in the assessment of alexithymia related to the developmental stages of emotional cognition and language. Biopsychosoc Med. (2009) 3:12. doi: 10.1186/1751-0759-3-12 19886981 PMC2777913

[10] Taylor G Ryan D. and Bagby M Toward the development of a new self-report alexithymia scale. Psychother Psychosom. (1985) 44:191–9. doi: 10.1159/000287912 3837277

[11] Sifneos PE. Alexithymia: past and present. Am J Psychiatry. (1996) . 153:137–42. doi: 10.1176/ajp.153.7.137 8659637

[12] Zimmermann G Rossier J Stadelhofen F Glillard F. Alexithymia assessment and relations with dimenssions of personality. Eur J psychol Assessment. (2005) 21:22–33. doi: 10.1027/1015-5759.21.1.23

[13] Erni T Lotscher K Modestin J. Two-factor solution of the 20-item Toronto Alexithymia Scale confirmed. Psychopathology. (1997) 30:335–40. doi: 10.1159/000285079 9444703

[14] Lane RD Ahern GL Schwartz GE Kaszniak AW. Is alexithymia the emotional equivalent of blindsight? Biol Psychiatry. (1997) 42:834–44. doi: 10.1016/S0006-3223(97)00050-4 9347133

[15] Wise TN Jani NN Kass E Sonnenschein K Mann LS. Alexithymia: relationship to severity of medical illness and depression. Psychother Psychosom. (1988) 50:68–71. doi: 10.1159/000288102 3255979

[16] Hendryx MS Haviland MG Shaw DG. Dimensions of alexithymia and their relationships to anxiety and depression. J Pers Assess. (1991) 56:227–37. doi: 10.1207/s15327752jpa5602_4 2056418

[17] Parker JD Bagby RM Taylor GJ. Alexithymia and depression: distinct or overlapping constructs? Compr Psychiatry. (1991) 32:387–94. doi: 10.1016/0010-440X(91)90015-5 1743009

[18] Ueno M Maeda M Komaki G. The different subgroups of high-scores on the TAS-20 based on the big five personality traits. Pers Individ Dif. (2014) 68:71–6. doi: 10.1016/j.paid.2014.04.012

[19] Arimura T Komaki G Murakami S Tamagawa K Nishikata H Kawai K. Development of the structured interview by the modified edition of Beth Israel hospital psychosomatic questionnaire (SIBIQ) in Japanese edition to evaluate alexithymia. Jpn J Psychosom Med. (2002) 42:259–69. doi: 10.15064/jjpm.42.4_259

[20] Taylor G Bagby M Parker J. Disorders of affect regulation: Alexithymia in medical and psychiatric illness. New York: Cambridge University Press (1997). doi: 10.1017/CBO9780511526831

[21] Bagby RM Taylor GJ Parker JD Dickens SE. The develpment of the toronto structured interview for alexithymia: item selection, factor structure, reliability and concurrent concurrent validity. Psychother Psychosom. (2006) 75:25–39. doi: 10.1159/000089224 16361872

[22] Grabe HJ Löbel S Dittrich D Bagby RM Taylor GJ Rufer M. The German version of the Toronto Structured Interview for Alexithymia: factor structure, reliability, and concurrent validity in a psychiatric patient sample. Compr Psychiatry. (2009) 50(5):424–30. doi: 10.1016/j.comppsych.2008.11.008 19683612

[23] Inslegers R Vanheule S SReiske M Virginie DMA Eline TMA Mattias D. Interpersonal problems and cognitive characteristics of interpersonal representations in alexithymia: a study using a self-report and interview-based measure of alexithymia. J Nerv Ment Dis. (2012) . 200:607–13. doi: 10.1097/NMD.0b013e31825bfad9 22759939

[24] Otsubo T Tanaka K Koda R Shinoda J Sano N Tanaka S. Reliability and validity of Japanese version of the Mini-International Neuropsychiatric Interview. Psychiatry Clin Neurosci. (2005) 59:517–26. doi: 10.1111/j.1440-1819.2005.01408 16194252

[25] Sheehan DV Lecrubier Y Sheehan KH Amorim P Janavs J Weiller E. The Mini-International Neuropsychiatric Interview (M.I.N.I.): the development and validation of a structured diagnostic psychiatric interview for DSM-IV and ICD-10. J Clin Psychiatry. (1998) 59 Suppl 20:22–33. 9881538

[26] Sriram TG Pratap L Shanmugham V. Towards enhancing the utility of Beth Israel Hospital Psychosomatic Questionnaire. Psychother Psychosom. (1988) 49:205–11. doi: 10.1159/000288085 3237971

[27] Krystal JH Giller ELJ Cicchetti DV. Assessment of alexithymia in posttraumatic stress disorder and somatic illness: introduction of a reliable measure. Psychosom Med. (1986) 48:84–94. doi: 10.1097/00006842-198601000-00007 3945719

[28] Costa PT McCrae RR. Personality and the five factor model of personality. J Person Disord. (1990) 4:362–71. doi: 10.1521/pedi.1990.4.4.362

[29] Shimonaka J Nakazato K Gondo K Takayama M. NEO-PI-R, NEO-FFI common manual(for Adults and college students). Tokyo: Tokyo psychology Co. Ltd (1999).

[30] Mehrabian A Epstein N. A measure of emotional empathy. J Pers. (1972) 40:525–43. doi: 10.1111/j.1467-6494.1972.tb00078.x 4642390

[31] Kato T Takagi H. A trait of the emotional empathy in adolescence Vol. 2. Tsukuba City: Stud Psychol Tsukuba Univ (1980) p. 33–42.

[32] Davis MH. Measuring individual differences in empathy: evidence for a multidimensional approach. J Pers Soc Psychol. (1983) 44:113–26. doi: 10.1037/0022-3514.44.1.113

[33] Aketa H. Structure and measurement of empathy: Japanese version of Davis’s interpersonal Reactivity Index (IRI-J). Psychol Rep Sophia Univ. (1999) 23:19–31. doi: 10.4992/jjpsy.88.15218

[34] Kojima M Furukawa TA Takahashi H Kawai M Nagaya T Tokudome S. Cross-cultural validation of the Beck Depression Inventory-II in Japan. (2002). Available online at: http://www.ncbi.nlm.nih.gov/entrez/query.fcgi?cmd=Retrieve&db=PubMed&dopt=Citation&list_uids=12127479. doi: 10.1016/S0165-1781(02)00106-3 12127479

[35] Nakazato K Mizuguchi K. Studies on psychometric characteristics of depression in the field of internal medicine. Jap J Psychosom Med. (1982) 22:107–12. doi: 10.15064/jjpm.22.2_107

[36] Spielberger C Gorsuch R Lushene R Vagg P Jacobs G. Manual for the state-trait anxiety inventory (STAI). Palo Alto, CA: Consult Psychol Press (1983).

[37] Shrout PE Fleiss JL. Intraclass correlations: uses in assessing rater reliability. psychol Bull. (1979) 86:420–8. doi: 10.1037/0033-2909.86.2.420 18839484

[38] Cole DA. Utility of confirmatory factor analysis in test validation research. J Consult Clin Psychol. (1987) 55:584–94. doi: 10.1037/0022-006X.55.4.584 3624616

[39] Marsh H Balla J McDonald R. Goodness-of-fit indexes in confirmatory factor analysis: The effect of sample size. Psychol Bull. (1988) 103:391–410. doi: 10.1037/0033-2909.103.3.391

[40] Browne MW Cudeck R. Alternative ways of assessing model fit. In: Bollen KA Scott Long J, editors. Testing structural equation models. Park Sage Publications, Newbury (1993). p. 136–62.

[41] Moriguchi Y Decety J Ohnishi T Maeda M Mori T Nemoto K. Empathy and judging other’s pain: an fMRI study of alexithymia. Hum Brain Mapp. (2009) 30:2063–76. doi: 10.1002/hbm.20653 PMC6871149 18781590

[42] Grynberg D Luminet O Corneille O Grèzes J Berthoz S. Alexithymia in the interpersonal domain: A general deficit of empathy? Pers Individ Dif. (2010) 49:845–50. doi: 10.1016/j.paid.2010.07.013

[43] Nemiah JC. Alexithymia. Theoretical considerations. Psychother Psychosomatics. (1977) 28:199–206. doi: 10.1159/000287064 609679

[44] Bird G Silani G Brindley R White S Frith U Singer T. Empathic brain responses in the insula are modulated by levels of alexithymia but not autism. Brain. (2010) 133:1515–25. doi: 10.1093/brain/awq060 PMC2859151 20371509

[45] Sekely A Bagby M Porcelli P. Assessment of the alexithymia construct. In: Luminet O Bagby RM Taylor GJ, editors. Alexithymia: advances in research, theory, and clinical rpactice. Cambridge University Press, New York (1997). p. 17–32.

[46] Honkalampi K DeBerardis D Velalante F Viinamaeki H. Relations between alexithymia and derpressive and anxiety disorders and personality. In: Luminet O Bagby RM Taylor GJ, editors. Alexithymia, advances in research, theory, and clinical practice. Cambridge: Cambridge Uiversity Press (2018). p. 142–73.

[47] Rosenberg N Rufer M Lichev V Ihme K Grabe H Kugel H. Observer-rated alexithymia and its relationship with the five-factor-model of personality. Psychologica Belgica. (2016) 56:118–34. doi: 10.5334/pb.302 PMC5854197 30479433

[48] Bach M Bach D Bohmer F Nutzinger DO. Alexithymia and somatization: relationship to DSM-III-R diagnoses. J Psychosom Res. (1994) 38:529–38. doi: 10.1016/0022-3999(94)90050-7 7990061

[49] Ahrens S Deffner G. Empical study of alexithymia; Methodology and results. Americal J Psychother. (1986) 40:430–47. doi: 10.1176/appi.psychotherapy.1986.40.3.430 3532835

[50] Meins E Fernyhough C Russell J. Security of attachment as predicter symbolic and mentalising abilities: A longitudianl study. Soc Dev. (1998) 7:1–24. doi: 10.1111/1467-9507.00047

[51] John OP Gross JJ. Individual differences in emotion regulation. In: Gross J, editor. Handbook of emotion regulation. Guilford Press, New York (2007). p. 351–72.

