# Supplementary material for: Development of a structured interview for the modified version of the Beth Israel Hospital psychosomatic questionnaire for alexithymia
Source: Front Psychiatry. 2024 Aug 2;15:1356643. doi: 10.3389/fpsyt.2024.1356643 (PMC11327022; doi:10.3389/fpsyt.2024.1356643)
Supplement: Supplementary file 1 [file Table_1.docx]

**Supplementary Materials**

**Twelve questions below show the sample questions and rating scales of *the Modified Version of the Beth Israel Hospital Psychosomatic Questionnaire Structured Interview (m-SIBIQ)***

***Interview Guidelines***

Explain the purpose of the interview to the participants and begin the interview once they agree with you. Ask them to freely discuss their current chief complaint(s) and history of present illness, then identify life events that may be associated with the onset or exacerbation of the disease(s) and complaints. Ask them how they feel about them.

Please include specific words that apply to your question. If they do not understand a question, explain it in a way that helps them understand. The interviewer may ask additional questions until the information necessary for the rating is obtained.

Regarding the rating criteria: An explanation of one's actions is not an expression of feelings or emotions. Expressions that appear unnatural and formal to the interviewer are inappropriate expressions of emotion.

For interviewing non-patient subjects (rather healthy persons), we modified the interview by adding questions about feelings and emotions in response to stressful events that had been experienced. If they replied that they had had no such life events, we asked them to imagine situations that typically evoke emotional responses, similar to the Alexithymia Provoked Response Questionnaire ([Krystal et al., 1986](#_ENREF_24)), and asked that they describe their own emotions.

1. **Feelings about chief complaints and/or exacerbating factors**

Depending on the answer to *Question 0*, ask *Questions 1*~ *8* or *Questions 9~ 12*, then evaluate the replies by using Questions *(#1)* through *(#7)* of *the m- SIBIQ.*

***Question 0***

**“Do you currently have any symptoms caused by an illness?” or “Have you ever suffered from poor health because of an illness?”, “When was that?”**

→ *If either answer is “Yes”*, proceed to *Question 1* below.

→*If the answer is “No”,* the interviewee is completely healthy and has no history of an illness. Proceed to *Question 9.*

***Question 1*:**

Ask about the current chief complaint (or the worst health condition in the past).

“Please tell me what is disturbing you now or disturbed you before?”

***Question 2:***

“Please tell me how you feel or felt about having your current or past chief complaint?”

*Pay attention to how they express feelings about the chief complaint.)

If they mention their feelings: ’

***Question 3:*** "Could you please explain how you feel in a little more detail?''

*About their medical history if they have one*;

***Question 4:*** “When did this (the patient’s current chief complaint) start?”

If the current chief complaint has occurred within the past year, identify the life events related to the onset. Identify life events related to the worsening of a chief complaint within the past year.

***Question 5*:** “Did anything change in your life when your current symptoms developed? For example, your work, school, family, or friends? How about the current chief complaint that occurred within the past year?

If their current chief complaint started more than a year ago but has gotten worse in the past year, identify life events related to the worsening of the chief complaint within the past year:

***Question 6:*** “Have the current symptoms gotten worse in the past year? When did that happen? Has anything changed in your life? For example, how about your work, school, family, or friends?

Ask how they felt about the life events identified in the question above.

***Question 7:*** “How did you feel about that?” (Focus on their emotional expressions. If the patient only describes the facts without describing feelings, ask the same question again.

If they mentioned their feelings or emotions;

***Question 8:*** “Could you please explain that in more detail.

II. When there are no physical complaints or symptoms:

***Question 9:***

Identify life events related to stress events.

"Do you suffer from any psychological stress due to anything like a difficult, exhausting, or sad past experiences?”

***Question 10:***

“When your symptoms occurred, was there any change in your life? For example, was there any change in your job, school life, family, or friends?”

→Ask how the interviewee felt about the event(s) identified above.

***Question 1I:***

“How did you feel about this?”

(Pay attention to emotional expressions; ask the same questions if the interviewee does not express emotions but only describes facts.)

If the interviewee mentioned an emotion:

***Question 12***

“Could you please elaborate a little more on that?

***Rating scale samples***

***m-SIBIQ (#1);***

*The interviewee mostly described the details of their complaint rather than his/her feelings.*

1 Not applicable (The interviewee described his/her feelings in detail but little about the complaint.)

2

3

4 More applicable than not (The interviewee described more about the complaint than about his/her feelings.)

5

6

7 Very applicable (The interviewee could not verbalize feelings at all, and only described his/her complaint.)

……………………….

***II. Recalling dreams and their details***

Ask the following question and evaluate the answer using questions (*#8*) and (*#10*) of *the m-BIQ*.

“Could you tell me about a dream you had recently?”

*Rating scale samples*

***m-SIBIQ (#8)*;**

The interviewee could recall the dream easily.

7 Not applicable (The interviewee could not recall any dreams.)

6

5

4 More applicable than not (The interviewee could recall a dream, but it took time to do it.)

3

2

1 Very applicable (The interviewee could recall a dream quickly.)

***m-SIBIQ (#10)*;**

Details of the interviewee’s dream were closer to daily thoughts or events than to being symbolic or abstract.

1 Not applicable (The dream was about events or incidents that were not actually experienced and was symbolic in nature.)

2

3

4 More applicable than not (close to daily events and thoughts.))

5

6

7 Very applicable (everyday events, mere reenactment of thoughts, no dreams)

**Ill Daydreaming, imaginative activities**

“You may fantasize or imagine things that would be enjoyable or fun to do in terms of hobbies, life, work,or school. For example, ``Going out with friends on Saturday and Sunday,'' “Buying clothes,” “Hoping to get a driver’s license soon,” or “Hoping to witb 10 million yen in the lottery.”

***m-SIBIQ (#11)*;**

The interviewee did not seem to have much re-imagination.

1 Not applicable (Enjoys daydreaming and imagining various things every day)

2

3

4 Somewhat applicable (Doesn’t daydream or imagine much)

5

6

7 Very applicable (Doesn’t daydream or imagine at all)

**IV Communicating feelings to others**

Ask if the interviewee can communicate their feelings to others and be understood.

“Have you ever talked about your feelings with your family, friends, or acquaintances?''

→If the interviewee answered yes to this:

"Please tell me about that experience?"

"Did you feel like your feelings were understood by talking to them like that?"

→If interviewee has never done this:

"Could you please tell me why?"

***m-SIBIQ (#12)*;**

The interviewee seemed able to easily share his feelings with others.

(Listen to specific conversations and objectively judge whether others empathize with the patient.)

7 Not applicable (Does not always get empathy when talking with others)

6

5

4 Somewhat true (Often talk to others and gain empathy from them)

3

2

1 Very applicable (Always feels empathy when talking with others)

***V　Expression of feelings to close people***

・“With your friends or family members can you tell them how you feel about them or the person you are dating?”

・If the interviewee mentions an emotion: "Could you please explain that in more detail?"

***m-SIBIQ (#9)*;**

The interviewee did not express feelings when talking about close friends or objects of affection (family members, lovers, etc.).

1 Not applicable (Expressed complex feelings in detail)

2

3

4 Somewhat applicable (Expressed feelings, but can't explain them in detail)

5

6

Very applicable (Was not able to express feelings.)

***Ⅲ. Fantasy and imaginary activities***

Ask the following question and evaluate the answer using Question (*#11*) of *m-SIBIQ*.

“Do you fantasize or imagine anything related to your hobby, life, or work (for a student something related to school) that would make you excited or happy if it……… ”

1 Not applicable (Does not seem to daydream or imagine much during the day.

2

3

4 Somewhat applicable (Does not daydream or imagine much )

5

6

7 Very applicable (Does not daydream or imagine at all.)

- *Original m-BIQ:* Twelve-item modified version of the Beth Israel Hospital Psychosomatic　Questionnaire. The original 7-point Lickert scales; from 1 (*Not applicable*) to 7 (*Very applicable*) ([Taylor, Bagby, & Parker, 1997](#_ENREF_1)).
